# Baseline predictors of treatment outcome in Internet-based alcohol interventions: a recursive partitioning analysis alongside a randomized trial

**DOI:** 10.1186/1471-2458-13-455

**Published:** 2013-05-07

**Authors:** Matthijs Blankers, Maarten WJ Koeter, Gerard M Schippers

**Affiliations:** 1 Jellinek, Arkin Mental Health Care, Amsterdam, The Netherlands; 2 Academic Medical Centre, Department of Psychiatry, Amsterdam Institute for Addiction Research, University of Amsterdam, Amsterdam, The Netherlands

**Keywords:** Alcohol, Internet, Intervention, Outcome predictors, RCT, Recursive partitioning

## Abstract

**Background:**

Internet-based interventions are seen as attractive for harmful users of alcohol and lead to desirable clinical outcomes. Some participants will however not achieve the desired results. In this study, harmful users of alcohol have been partitioned in subgroups with low, intermediate or high probability of positive treatment outcome, using recursive partitioning classification tree analysis.

**Methods:**

Data were obtained from a randomized controlled trial assessing the effectiveness of two Internet-based alcohol interventions. The main outcome variable was treatment response, a dichotomous outcome measure for treatment success. Candidate predictors for the classification analysis were first selected using univariate regression. Next, a tree decision model to classify participants in categories with a low, medium and high probability of treatment response was constructed using recursive partitioning software.

**Results:**

Based on literature review, 46 potentially relevant baseline predictors were identified. Five variables were selected using univariate regression as candidate predictors for the classification analysis. Two variables were found most relevant for classification and selected for the decision tree model: ‘living alone’, and ‘interpersonal sensitivity’. Using sensitivity analysis, the robustness of the decision tree model was supported.

**Conclusions:**

Harmful alcohol users in a shared living situation, with high interpersonal sensitivity, have a significantly higher probability of positive treatment outcome. The resulting decision tree model may be used as part of a decision support system but is on its own insufficient as a screening algorithm with satisfactory clinical utility.

**Trial registration:**

Netherlands Trial Register (Cochrane Collaboration): NTR-TC1155.

## Background

Harmful alcohol use is a major contributor to the global burden of disease [1] and is considered to be the main cause of nearly 4% of global mortality [2]. The magnitude of this burden partly results from the wide treatment gap, which represents the difference between the prevalence of harmful alcohol use and the number of individuals receiving treatment for harmful alcohol use [3]. The development and use of innovative treatment options (e.g. Internet-based interventions) could narrow the treatment gap for harmful alcohol use.

Internet-based interventions are seen as an attractive option for people who meet harmful alcohol use criteria and who have relatively mild conditions [4-7]. Moreover, these interventions have been found effective in addressing harmful drinking behaviour and improving quality of life (e.g. [7-9]; for a review see: [10]). There are also indications that Internet-based alcohol interventions are cost-effective [11].

However, there is notable heterogeneity in treatment outcomes, which several recently published studies have demonstrated. Postel and colleagues [9] found that three months after baseline, 32% of the alcohol E-therapy participants had not reached a drinking level within the British Medical Association (BMA) drinking guideline limits (no more than 21 standard glasses per week for men, 14 standard glasses per week for women). Riper et al. [7] conclude that after six months, the majority (83%) of the participants in their ‘Drinking Less’ Internet-based self-help program still consumed more alcohol than the BMA guideline recommends. A study by our research group [8] found that 71% of the self-help program participants had an unsuccessful treatment outcome six months after baseline.

A number of studies have explored clinical outcome predictors of face-to-face alcohol therapy. These studies have studied the predictive potential of a large number of possible baseline predictors regarding alcohol consumption, other substance use, psychosocial functioning, and demographic characteristics. The research results are mixed: while some authors have identified relevant predictors, other authors have not been able to replicate this. Adamson and colleagues concluded in a recent review [12] that attempts to synthesize findings on patient predictors of alcohol treatment outcome were also hampered by lack of agreement of the best measure for predictor variables.

For the purpose of the current study, a literature search on PubMed / MEDLINE (1980–2011) using as search term the title words (alcohol OR drink* OR substance *use*) AND (predict* OR outcome* OR treatment) resulted in 5041 articles. The abstracts of potentially relevant articles were screened and those that were considered relevant for our literature overview were retrieved, to identify studies in which the same baseline and/or outcome variables were used as available in our dataset). Based on expert advice, 5 more articles were added to our literature database. A brief overview of the findings reported in 17 publications with the highest relevance to our literature review is presented below.

A number of studies have found a negative relationship between the severity of drinking problems at baseline and clinical outcome [13-15]. McKay & Weiss [16] however report a positive relationship between baseline drinking problems and clinical outcome. Age of first alcohol consumption, overall duration of alcohol problems and number of previous quit attempts have been linked to treatment outcome [13]. With regard to psychosocial functioning, several measures have been found to predict intervention outcome: self-efficacy [17,18], motivation to change [18-21], internal locus of control, coping skills, low levels of experienced stress, concern from partners or peers, and a stable social environment [13,16,22-24]. Social problems and psychopathology are found to negatively correlate with successful outcome [13,16,22,25]. Particular demographic characteristics, such as age, sex, education level, being of foreign origin, and general socioeconomic status have been linked to clinical outcome [18,22,26,27], although these findings have not always been replicated [16,22,28]. To date, only one paper by Riper and colleagues [29] has assessed which baseline variables predict clinical outcome in Internet-based alcohol interventions. The authors concluded that being female and highly educated were correlated with receiving benefits from an Internet-based self-help intervention.

All in all, it is difficult to define a core set of predictors that should be included in a model aiming to predict treatment outcome. Thus, a large number of possible predictors will be considered for inclusion in the current analysis. Interactions between the possible predictors will also be taken into account, with the aim to test whether a valid predictive model, which can be used as a screening or decision-support tool, can be found. While it is generally assumed that a large sample size will be needed in order to construct and test a model comprising a large number of predictors (and possibly an even larger number of interactions among these predictors), this is not necessarily true [30]. In the current study, a classification tree analysis will be performed using recursive partitioning. Using this data-driven technique, it is feasible to analyze multi-dimensional data in a dataset with a limited sample size [31]. This is an important advantage of recursive partitioning over generalized linear modelling regression analysis. Recursive partitioning can be used to identify variables that are of relevance to future research, but also to create data-driven, evidence-based treatment decision support tools [30]. For example, Swan and colleagues [32] identified relevant variables when examining the heterogeneity of their outcomes from a smoking cessation intervention using recursive partitioning. Others [33] have used recursive partitioning in an analysis of pregnant women’s responses to substance use questions, which resulted in a three-item Substance Use Risk Profile-Pregnancy scale. In the current study, recursive partitioning is used in an analysis of data from a randomized controlled trial (RCT) performed in the Netherlands, comparing the effectiveness of Internet-based therapy and Internet-based self-help for harmful alcohol use. Results from this study have been published elsewhere [8]. The current analysis will be performed in order to test whether a screening instrument with acceptable sensitivity and specificity can be developed.

## Methods

### Ethics statement

The source of the data for the present analysis comes from a trial registered in the Netherlands Trial Register (Cochrane Collaboration), and is traceable as NTR-TC1155. This trial was conducted in compliance with the Helsinki Declaration and was approved by the Medical Ethics Committee of the University of Amsterdam, Academic Medical Centre. All participants provided written informed consent online and were provided with contact information of the collaborating treatment centre, the researchers, and an independent physician (in case they wanted additional treatment).

### Participants

**Figure 1:**
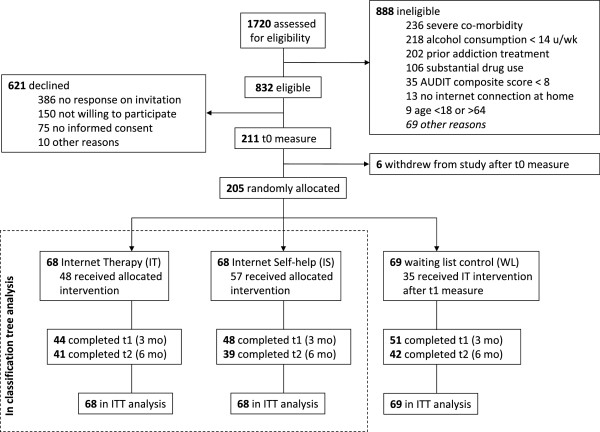
CONSORT trial flow diagram.

Participants were recruited through the participating substance abuse treatment centre (SATC) in Amsterdam, the Netherlands, between June 2008 and June 2009. Participants were randomly allocated to one of three arms of the trial: Internet therapy (IT), Internet self-help (IS) or non-treated waiting list (WL) (Figure 1). In the RCT, 205 participants were included, 68 in the IT arm, 68 in the IS arm, and 69 in the WL arm. In the analysis reported here, only the data from participants of IT (*n* = 68) and IS (*n* = 68) are analyzed (Table 1). The sample consisted of equal proportions men (49%) and women (51%). On average, they were 41.5 (SD = 9.8) years of age. They consumed an average of 44.3 (SD = 25.2) standard glasses of alcoholic beverages (10 *g* ethanol) per week at baseline. The combined quantity of alcohol consumption and an average Alcohol Use Disorders Identification Test (AUDIT) composite score of 19.2 (SD = 5.2) indicated that the participants demonstrated unhealthy drinking behaviours at baseline. Over 80% of the participants were employed, and about 50% had obtained a high level of education. The majority of the participants from the study lived in an urban setting, and all participants were living in the Netherlands. Baseline sample characteristics were evenly distributed over the two interventions (IT and IS). Success rates for IT where somewhat higher than for IS (53% versus 29%, Fisher’s exact test = 7.771, *p* = 0.009) at six months post-randomization. Relatively large effects of therapist involvement in Internet-based interventions for harmful alcohol use are reported by others as well [9], although others sometimes find no association between therapist involvement and outcome [10]. On average, 41% of the participants in this study had successfully responded to treatment. The effect sizes for Internet-based therapy and Internet-based self-help in this study were in line with what others report in the literature (e.g. [7,9]).

**Table 1:** Sample characteristics

**Variable**	**IT (*n *= 68)**	**IS (*n *= 68)**	***t *or Fisher’s exact**	***p***
Women	35 (51%)	35 (51%)	0.000	1.000
Age (years)	41.9 (10.1)	41.1 (9.6)	0.487	0.627
Education			4.494	0.103
Low	2 (3%)	7 (11%)		
Medium	24 (38%)	30 (46%)		
High	38 (59%)	29 (44%)		
Employed	58 (85%)	55 (82%)	0.254	0.648
Residential urbanization level			0.744	0.748
Low	9 (13%)	6 (9%)		
Medium	21 (31%)	22 (32%)		
High	37 (55%)	40 (59%)		
AUDIT composite score	18.8 (4.8)	19.6 (5.6)	0.977	0.330
Years of alcohol problems	5.2 (5.7)	5.4 (5.7)	0.225	0.823
Drinks per week	45.2 (26.3)	43.4 (24.0)	0.379	0.706
Drinking days per week	6.0 (1.5)	5.6 (2.1)	1.392	0.166
Cannabis lifetime use	29 (43%)	21 (31%)	2.024	0.213
Cocaine lifetime use	17 (25%)	11 (16%)	1.619	0.289
Amphetamine lifetime use	14 (21%)	12 (18%)	0.190	0.828
QOLS composite score	73.1 (14.4)	71.5 (20.0)	0.541	0.589
EQ-5D score	0.79 (0.20)	0.80 (0.18)	0.316	0.752
BSI global severity index	0.81 (0.49)	0.77 (0.52)	0.531	0.597
Treatment response (6 months)	36 (53%)	20 (29%)	7.771	0.009

Presented data are counts (%) or mean (SD); Education classification according to International Standard Classification of Education Standard [34]; AUDIT = Alcohol Use Disorders Identification Test; QOLS = Flanagan Quality of Life Scale; EQ-5D = 5 dimensional EuroQol instrument, score calculated using the MVH-A1 algorithm from Dolan [35]; BSI = Brief Symptom Inventory.

### Procedure and interventions

Participants were invited to complete the online baseline assessment if they met inclusion criteria and provided informed consent. After completion of the baseline assessment, participants were randomly allocated to one of the trial arms. Participants in the IS arm participated in a stand-alone, Internet-based, non-therapist involved, fully automated, self-guided treatment program, based on a cognitive behavioural therapy (CBT) and motivational interviewing (MI) treatment protocol [36]. IS introduced participants to CBT treatment exercises in order to help them modify their alcohol consumption. These treatment exercises (1) prompted the participant to report their alcohol consumption, the context in which they consumed alcohol, and their inner state at the time of alcohol consumption; and (2) compared the participants’ current alcohol consumption levels with the drinking goal the participant had set. By doing the exercises, participants acquired skills and knowledge about coping with craving, drinking lapses, peer pressure, and how to stay motivated in high-risk situations. Participants allocated to IT participated in Internet-based therapy, based on the same CBT/MI treatment protocol as IS. IT used the same treatment exercises as IS, but included up to seven synchronous text-based chat-therapy sessions lasting 40 min. each. At the start of IT, each participant was assigned to a therapist. The therapists all had a bachelor’s or a master’s degree in psychology, were trained in CBT and supervised by Ph.D.-level psychologists, and worked for the collaborating treatment centre.

All RCT participants were invited for a follow-up assessment at three and six months post-randomization. As attrition rates for Internet-based RCTs are often high compared to other types of RCT interventions [37], extra effort was made to maximize response and retention rates. This was done by offering participants a remuneration for assessment completion (€15 gift coupons per completed assessment), sending email reminders, and contacting participants via telephone if necessary to motivate them to fill out the Internet-based follow-up assessments. If a participant was unresponsive to these encouragements, data collection was done by telephone. This resulted in response rates of 70% and 60% at three and six months post-randomization, respectively. At both the 3-month and the 6-month assessment, there were no significant differences between the two methods (Internet or telephone) of data collection in terms of the number of drinks that participants reported having consumed during the 7 days before the assessment: at 3 months (M = 30.3, SD = 24.7 vs. M = 28.0, SD = 22.0), *t*(203) = 0.554, *p* = 0.58; at 6 months (M = 31.2, SD = 25.2 vs. M = 28.2, SD = 22.2), *t*(203) = 0.524, *p* = 0.60.

### Measures

Baseline predictors were collected prior to randomization for all included participants. Based on the literature search on predictors of treatment outcome presented in the Background section, three categories of predictor variables were formed. Category A – Substance abuse variables, contained 12 predictors, including scores from the AUDIT [38], an alcohol use disorders screening instrument with favourable psychometric properties [39]; standard drinking units consumed per drinking day; drinking days per week; duration (years) of alcohol problems; and use of illegal substances. Category B measured psychosocial functioning and included 27 predictors, such as quality of life (measured with the Quality of Life Scale, QOLS [40] and EuroQol, EQ-5D [41]); subscales of the Brief Symptom Inventory (BSI) [42]; and items from the Working Ability Index (WAI) [43]. The QOLS has been found to be a valid instrument for measuring domains of quality of life across diverse patient groups [44]. The EQ-5D is widely used as a quality of life instrument in mental health and addiction research. Günther and colleagues found moderate support for its validity, although the EQ-5D showed a moderate ceiling effect [45]. The BSI was developed from the SCL-90-R, and psychometric evaluation indicates it is an acceptable short alternative. Both test-retest and internal consistency reliabilities are very good. High convergence between BSI scales and like dimensions of the Minnesota Multiphasic Personality Inventory provide good evidence of convergent validity [42]. The WAI is found to be internally coherent and considered a very predictive and cross-nationally stable instrument [46]. Category C contained 7 demographic characteristics including: sex, age, education level, urbanisation level in place of residence, and living situation (alone / shared). The dependent variable was treatment response, six months post-randomization. Treatment response is based on alcohol consumption during the past 7 days, and defined in the RCT study protocol as (1) alcohol consumption within the British Medical Association boundaries (no more than 14 standard units for women, or 21 units for men, per week) [47], and (2) no deterioration of more than 10% on the AUDIT [39], the QOLS [40] or the global severity index of the BSI [42]. In other words, any such deterioration, or drinking more than the British Medical Association boundaries, precludes treatment response. Positive treatment response should be interpreted as having a desirable outcome of treatment, covering the wider aspects of problem drinking beyond the quantity of alcohol consumption alone [48].

### Statistical methods

First, all of the 46 potential predictors were run through a univariate regression analysis, with the dependent variable treatment response 6 months post-randomization. Only potential predictors with a *p*-value of ≤ 0.15 in the univariate regression analysis were selected as predictors for the recursive partitioning analysis. The *p* ≤ 0.15 level was chosen based on work by Bendel & Affifi [49] on stepwise regression. They suggest that a *p* ≤ 0.05 level will often exclude important variables from the model. Although a higher *p*-value limit raises the risk of Type I error, it reduces the risk of not finding a relationship between variables that is really there (Type II error), something that would be especially regrettable in exploratory research. Other researchers support the suggestion by Bendel & Affifi to raise the *p*-level for predictor selection based on univariate analysis [50,51].

### Recursive partitioning

Recursive partitioning is a non-parametric regression approach; its main characteristic is that the space spanned by all predictor variables is recursively partitioned into a set of areas. A partition is created such that observations with similar response values, or (as in this case) participants with similar treatment outcome are grouped together. After the partitioning is completed, a constant value of the response variable is predicted within each area [52]. As a result, recursive partitioning examines all available predictors and identifies a series of variables that are most related to the outcome measure. It is an exploratory technique, and yields results that are easily interpretable and usually presented in classification trees. Zhang and Singer [53] have published an overview of recursive partitioning methods, classification trees, and applications.

In this study, recursive partitioning was performed using the computational package *party*[54] version 0.9-9999 for the *R* statistical environment version 2.11.1 [55]. The *party* package is a computational toolbox for recursive partitioning. The core of the package is an implementation of conditional inference trees which embed tree-structured regression models into a well defined theory of conditional inference procedures. This non-parametric class of regression trees is applicable to all kinds of regression problems, including nominal, ordinal, numeric, censored as well as multivariate response variables and arbitrary measurement scales of the covariates [54]. For this analysis, the minimum criterion for making a split in the classification tree was set at *p* = 0.15, in line with the *p*-value chosen based on [49] for the inclusion of variables in the recursive partitioning analysis after univariate analysis. The minimum number of participants in a subgroup was set at *n* = 25. Even for small samples, a value of 20–30 is often chosen as a minimum subgroup size, in order to prevent over-fitting. Smaller subgroups often have to be pruned away in the cross-validation [56]. In order to assess the stability of the classification trees obtained, trees were calculated using the original, complete (*n* = 136) dataset, but also on 100 resampled datasets of *n* = 135, created using a leave-one-out jackknife approach. The resulting 100 jackknife trees were compared to the initial (*n* = 136) tree.

In a consecutive step, the predictive validity of the classification tree with regard to treatment response was assessed. By comparing the accuracy of the classification tree with random classification, the improvement in predictive accuracy of applying the classification tree was assessed. Confidence intervals were estimated by creating 200 bootstrapped samples from the original dataset on which the calculations were performed.

To deal with missing data resulting from non-response, the imputation software Amelia-II was used. In a simulation study that used data collected in the pilot study of this RCT, it was confirmed that the use of Amelia-II for imputing missing observations in longitudinal datasets containing non-normal distributed alcohol count data led to accurate results [57]. For the current study, a single imputation for the missing observations in the dataset was created. The significance level for all analyses was set at *α* = 0.05, and all analyses were carried out using the *R* software environment for statistical computing version 2.11.1 [55].

## Results

### Identification of possible predictors

Associations between the ‘treatment response’ outcome and all 46 predictors were explored. This resulted in the identification of five predictors with *p* ≤ 0.15: (a) *drinking days per week*; BSI subscales (b) *interpersonal sensitivity* and (c) *hostility*; (d) *cognitive working ability*; and (e) *living alone*.

### Classification analysis

**Figure 2:**
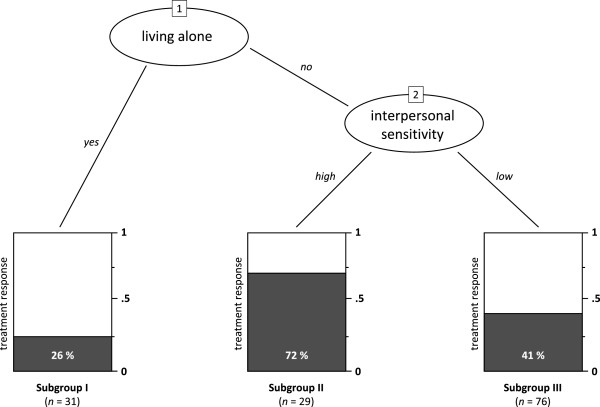
**Recursive partitioning classification tree analysis of treatment response to Internet-based alcohol interventions six months after baseline.** Subgroup I (*n* = 31): Living alone; Subgroup II (*n* = 29): not living alone, high interpersonal sensitivity; Subgroup III (*n* = 76): not living alone, low interpersonal sensitivity.

The recursive partitioning identified three subgroups from the five predictors detected by the univariate regression analysis. These three subgroups differed in their predicted probability of a positive treatment outcome. Results demonstrated that the optimal split in three subgroups could be made using two of the five predictor variables, *living alone* and *interpersonal sensitivity*. Figure 2 shows the classification analysis outcomes of the 136 participants in the sample. In the ovals of this figure, the two splitting variables are presented. In the first step (oval 1), 31 of 136 participants reported that they were living alone at baseline. This group of participants (Subgroup I: living alone) had a relatively low probability (0.26) of treatment response at six months post-randomization. In the second step (oval 2 of Figure 2), the remaining 105 participants were split into two groups based on their score on interpersonal sensitivity (BSI subscale). Twenty-nine participants (Subgroup II: not living alone, high interpersonal sensitivity) scored relatively high (at least 1) on interpersonal sensitivity. This subsample had a high probability of 0.72 on treatment response. The other 76 participants (Subgroup III: not living alone, low interpersonal sensitivity) had an intermediate probability (0.41) on treatment response. Fisher’s exact test confirmed that the proportion treatment response differed between Subgroup I and II (*p* = 0.0006), Subgroup II and III (*p* = 0.005), but not between Subgroup I and III (*p* = 0.19). In Table 2, information on baseline characteristics and treatment response in the three subgroups is presented. Some items differed significantly between the three subgroups created through partitioning. These items included the dependent variable treatment response, and all but one of the variables selected in the univariate regression analysis.

**Table 2:** Treatment response and odds ratios for the three subgroups resulting from classification tree analysis

	**Subgroup**				
***Characteristic***	**I (*n *= 31)**	**II (*n *= 29)**	**III (*n *= 76)**	**F / Fisher’s exact**	***p***
Living alone	31 (100%)	0 (0%)	0 (0%)	134.564	0.000*
Interpersonal sensitivity	0.98 (0.63)	1.83 (0.53)	0.52 (0.32)	85.548	0.000*
Hostility	0.68 (0.59)	1.04 (0.67)	0.48 (0.42)	11.863	0.000*
Cognitive working ability	3.40 (0.77)	3.07 (0.88)	3.68 (0.70)	7.028	0.001*
Treatment response	8 (26%)	21 (72%)	31 (41%)	13.884	0.001*
IT intervention	14 (45%)	16 (55%)	38 (50%)	0.618	0.716
Treatment response (OR [95% CI])	0.50 [0.20, 1.27]	3.81 [1.50, 9.67]	*1*		
Women	16 [52%]	15 (52%)	39 (51%)	0.038	1.000
Age	41.5 (11.4)	40.7 (9.4)	41.8 (9.4)	0.140	0.870
Education				4.468	0.336
Low	2 (7%)	4 (14%)	3 (4%)		
Medium	15 (50%)	11 (39%)	28 (39%)		
High	13 (43%)	13 (46%)	41 (57%)		
Employed	24 (77%)	23 (82%)	66 (87%)	1.662	0.413
Residential urbanization level				3.923	0.419
Low	3 (10%)	5 (17%)	7 (9%)		
Medium	7 (23%)	11 (38%)	25 (33%)		
High	21 (68%)	13 (45%)	43 (57%)		
AUDIT composite score	18.5 (5.8)	20.9 (4.4)	18.9 (5.2)	1.859	0.160
Years of alcohol problems	4.2 (4.7)	6.6 (6.8)	5.3 (5.6)	1.291	0.278
Drinks per week	40.6 (25.9)	48.1 (23.5)	44.2 (25.4)	0.666	0.516
Drinking days per week	5.7 (2.0)	5.9 (1.7)	5.8 (1.8)	0.059	0.943
Cannabis lifetime use	11 (36%)	8 (28%)	31 (41%)	1.556	0.443
Cocaine lifetime use	2 (7%)	8 (28%)	18 (24%)	5.512	0.061
Amphetamine lifetime use	3 (10%)	6 (21%)	17 (22%)	2.335	0.323
QOLS composite score	69.8 (17.0)	64.4 (15.2)	76.4 (17.3)	5.726	0.004
EQ-5D score	0.76 (0.23)	0.72 (0.27)	0.84 (0.12)	4.859	0.009
BSI global severity index	0.88 (0.48)	1.39 (0.40)	0.53 (0.31)	47.790	0.000*

Presented data are counts (%) or mean (SD) unless indicated otherwise; OR [95% CI] indicates odds ratios and their respective 95% confidence interval [lower, upper]. Subgroup III is the reference category; Education classification according to International Standard Classification of Education standard [34]; AUDIT = Alcohol Use Disorders Identification Test; QOLS = Flanagan Quality of Life Scale; EQ-5D = 5 dimensional EuroQol instrument, score calculated using the MVH-A1 algorithm from Dolan [35]; BSI = Brief Symptom Inventory; * Significant at *α* = 0.05 level, after Bonferroni correction for 21 variables in this table: corrected *α* = 0.05 / 21 = 0.0024.

### Robustness of the classification trees

Using leave-one-out jackknife resampling, the robustness of the presented classification tree was assessed. One hundred jackknife resamples were created. For each created resample, the data from one random participant was left out of the analysis. After each of the resample iterations, the construction of the classification tree was replicated based on the data from the remaining 135 participants. If the proposed classification tree was robust, the same tree presented in Figure 2 would show up in most of the 100 iterations. If the classification tree was not robust and sensitive to small random changes in the data, a variety of different trees would result as a consequence of minor data variations due to resampling. Under the current conditions, 67 out of 100 classification trees based on the jackknife resampled datasets were identical to the tree presented in Figure 2 (i.e. variables, order of variables, and tree splits were the same). All of the 100 generated trees selected the *living alone* variable as the first splitting variable. All 100 regenerated trees were constructed with two variables, with the second variable splitting the *shared living* subsample in two (as is the case in the presented figure). There was variability in the second splitting variable selected. In 67 / 100 iterations *interpersonal sensitivity* was selected, in 25 / 100 *hostility*, and in 8 / 100 *cognitive working ability*.

### Predictive validity of the classification tree

In order to estimate the predictive validity of the presented classification tree for new data, 200 bootstrap resamples with *n* = 136 of the original dataset were created. For each of the bootstrap resamples, the classification tree was used to predict whether a participant had a low or high probability of treatment response, six months post-randomization. The predictions made using the classification tree were compared to a 50% random chance model with regard to the number of correctly classified participants (Table 3).

**Table 3:** Performance characteristics of the predictors in the classification trees with 95% confidence intervals

**Classification**	**Sensitivity**	**Specificity**	**Negative predictive value**	**Positive predictive value**
Chance (random classification)	0.50 [0.40, 0.58]	0.50 [0.43, 0.58]	0.56 [0.46, 0.66]	0.45 [0.33, 0.56]
Screener conservative	0.34 [0.21, 0.48]	0.89 [0.82, 0.96]	0.63 [0.53, 0.73]	0.72 [0.54, 0.88]
Screener progressive	0.87 [0.74, 0.93]	0.30 [0.19, 0.39]	0.73 [0.54, 0.86]	0.49 [0.39, 0.58]

Bootstrapped (200 iterations) 95% confidence intervals are displayed within brackets [lower, upper]; Screener conservative interprets subgroup III as responding negative to treatment; Screener progressive interprets subgroup III as responding positive to treatment; Sensitivity is the proportion of actual positive treatment responders which are correctly identified; Specificity is the proportion of negative treatment responders which are correctly identified; Negative predictive value is the proportion of participants with negative predicted outcome who are correctly identified; Positive predictive value is the proportion of participants with positive predicted outcome who are correctly identified.

A 50% random chance model has both sensitivity and specificity values of 0.5. Two different screener algorithms are proposed in Table 3, depending on how Subgroup III (probability of 0.41 on treatment response) is interpreted. In the conservative screener algorithm, Subgroup III is predicted unresponsive to treatment. This is conservative in the sense that the risk is low in wrongfully predicting that a participant will have treatment success when in actuality (s)he will not. This conservative assumption, however, has a price: a relatively large proportion of treatment responders are wrongfully classified as non-responders. In the progressive screener algorithm, Subgroup III is predicted to show treatment response. The risk of wrongfully predicting that a participant is a treatment responder when (s)he is not is high when using this screener. On the other hand, there are not many treatment responders that will be misclassified based on the more progressive of the two proposed screening algorithms. Compared to the random chance model, the algorithm based on recursive partitioning had either higher specificity (0.89) with lower sensitivity (0.34) (conservative, Subgroup III predicted to be treatment non-responder), or lower specificity (0.30) with higher sensitivity (0.87) (progressive, Subgroup III predicted to be treatment responder). Differences in the same direction appeared for the negative / positive predictive value. Where the 95% confidence intervals for the three classification tree models overlap, the differences are not statistically significant.

## Discussion

The most relevant classification variables to predict treatment outcome (6 months post-randomization) were whether a participant lived alone (*living alone*) and *interpersonal sensitivity* (measured in a subscale of the BSI). Participants living alone had a relatively low probability of positive treatment outcome, whereas participants who lived with others and scored high on interpersonal sensitivity had a relatively high probability of positive treatment outcome. Participants in a shared living condition and low score on interpersonal sensitivity had a moderate probability of positive treatment outcome. With the exception of the BSI global severity index, the three subgroups did not differ significantly on any of the other baseline measures, after Bonferroni correction.

It is remarkable that from 46 predictors found in the literature, only five remain candidate predictors for the recursive partitioning procedure after univariate regression analysis. The exclusion criterion for predictors (*p* ≥ 0.15) can even be considered lenient. Against a conventional significance level of *α* = 0.05, *living alone* would have been the only significant predictor (*p* = 0.02) out of the 46 tested predictors. This indicates that either the dataset in this analysis is different from other harmful alcohol use treatment datasets used to explore outcome predictors (e.g. due to a difference between face-to-face and Internet-based interventions), or it might indicate methodological flaws in some other studies (e.g. insufficient correction for multiple testing which would result in many false positive test results in explorative studies).

The reported results were moderately robust against small fluctuations in the sample based on which the classification tree was constructed. The classification tree predicts above chance level: when making conservative assumptions, the instrument has a high specificity, and when the assumptions are more progressive, a high sensitivity is obtained. However, the utility of this screening instrument as a stand-alone decision tool is limited, considering the low sensitivity under the conservative assumption, and the low specificity under the progressive assumption.

### Limitations

The results of this study should be considered in light of its limitations. Only those limitations related to the current recursive partitioning analysis will be discussed; limitations regarding the RCT and interpretation of its clinical results have been discussed elsewhere [8].

The sample size in the RCT had sufficient power to draw conclusions for the main research questions (regarding effectiveness of the interventions). However, for secondary explorative analyses of subgroups performed in the current study, the sample size was somewhat small. Although recursive partitioning does not use significance tests (and therefore no concept of power applies to guide a power or sample size analysis) [58], it is generally conceived that a sample size of 100–150 is the minimum for making recursive partitioning worth attempting [59]. Based on this view, the sample size of *n* = 136 in the current study is just about the required minimum. In order to achieve this sample size, data from IT and IS participants had to be pooled. The underlying assumption of this pooling is that the relation between predictors and outcome is comparable for these two interventions. Some support for this assumption may be inferred from results from project MATCH. This project was an 8-year, multi site, $27-million investigation that studied which types of alcoholics respond best to which forms of treatment. The results with regard to patient matching in this study suggest that triaging clients to a particular treatment, at least based on the attributes and treatments studied in project MATCH, will not assure treatment success as previously believed [60]. This means that the baseline matching variables of the project MATCH sample do not differentiate between which form of treatment will be most effective for a specific client, and thus that the relation between these predictors and outcome is comparable for the different interventions. To what extend these findings in project MATCH can be transferred to low-intensity Internet-based alcohol interventions is a matter of debate.

Recursive partitioning is primarily a data driven approach. Debate remains as to whether recursive partitioning is prone to over-fitting the data or not. Either way, the resulting classification tree is always one of the possible solutions rather than the only solution to fit data. However, as in this study a univariate regression analysis was performed to empirically support the selection of candidate predictor variables, the current classification tree was the only possible solution when following this procedure. Another common critique on recursive partitioning is its sensitivity to small changes in the data used. The robustness of the presented model is assessed based on a resampling approach and was found to be moderately stable. A methodologically stronger approach would have been to use two separate datasets, the first to construct the classification tree, and the second to evaluate the model and to calculate the statistics presented in Tables 2 and 3. Therefore a validation of the model in a new sample would be desirable before future use of the presented model is considered.

The current study is performed using data from only one trial on Internet-based alcohol interventions, and in this trial, many of eligible participants refused to participate. Although compared to all 832 people who were eligible to participate, the participants who were included reported somewhat higher, but not significantly higher baseline AUDIT scores (*p* = 0.11, see [8]), generalizations beyond this study population are only possible to a limited extent. A number of factors may play a role in successful outcome resulting from an intervention. Treatment outcome itself is one of these factors, however other factors impact a participant’s recovery process over time. This study did not allow for disentanglement of treatment effects and other effects (e.g. natural recovery rates). Given this fact, the current classification tree should in no way be regarded as a causal model of treatment response, but merely as the unique outcome of the recursive partitioning approach taken in combination with the current dataset.

### Strengths

The main strength of the study is the thorough statistical approach. A selection of possible predictors was made based on the literature on outcome predictors in alcohol treatment studies. The recursive partitioning software was used in such a manner that the formulation of small, clinically irrelevant subgroups was prevented. The robustness of the presented tree (Figure 2) was tested using a leave-one-out jackknifing approach, in which it was shown that in the majority of the resampled datasets, the same classification tree was formed. The presented classification tree was tested by classifying actual cases in the bootstrapped samples of the dataset.

### Implications and future research

If the results presented in this paper find themselves replicated or extended by future studies, this ultimately could lead to the development of an evidence based intervention allocation decision support system. This system could be helpful for problem drinkers contemplating whether participation in an Internet-based intervention would be profitable to them. Currently, it is often reported that Internet-based interventions lead to a favourable treatment outcome for some of the participants, but not for others. This is also the case for face-to-face addiction treatment. An instrument with predictive validity to profile those that will likely have favourable treatment outcome after addiction treatment interventions is therefore highly needed [61]. The results of the current study contribute to the development of such an instrument for Internet-based alcohol interventions.

However, before the two variables living alone and interpersonal sensitivity can be used in a profiling instrument, a necessary step for future research would be to test the presented model on an alternative dataset. Although the robustness of the presented tree has been tested using resampling in this study, applying the tree on a different dataset (but with the predictor and outcome variables measured) would further support its validity if the predictive validity of the two variables can be replicated. If this validation would be successful, an advantage of the two currently presented predictors (living alone, interpersonal sensitivity) is that they are easy to measure as they are based on only a few self-report items, which would make the development of a self-report decision support system more practically feasible.

## Conclusions

This study demonstrated how baseline variables can be used to construct a classification tree assessing a participant’s probability of treatment response. The algorithm presented in this paper may be used to support clinical decision-making, but should not be used without careful reflection to determine who should and should not be provided Internet-based treatment. Harmful drinkers in a shared living situation, with a high score on interpersonal sensitivity, have a significantly higher probability of treatment response in Internet-based alcohol interventions compared to other participants in different contexts and scores.

## Competing interests

The authors declare that they have no competing interests.

## Authors’ contributions

MB, MWJK, GMS designed the RCT from which the data reported in this article stems; MB, MWJK, GMS conceived the study presented in this manuscript. MB, MWJK planned and performed the statistical analysis for this manuscript; MB, MWJK, GMS drafted the submitted manuscript. All authors read and approved the final manuscript.

## Pre-publication history

The pre-publication history for this paper can be accessed here:

http://www.biomedcentral.com/1471-2458/13/455/prepub
